# RNase E in the γ-Proteobacteria: conservation of intrinsically disordered noncatalytic region and molecular evolution of microdomains

**DOI:** 10.1007/s00438-014-0959-5

**Published:** 2014-11-29

**Authors:** Soraya Aït-Bara, Agamemnon J. Carpousis, Yves Quentin

**Affiliations:** 1 Laboratoire de Microbiologie et Génétique Moléculaires, UMR 5100, Centre National de la Recherche Scientifique and Université Paul Sabatier, 118, route de Narbonne, 31062 Toulouse Cedex 9, France; 2 Present Address: Microbes, Intestin, Inflammation et Susceptibilité de l’Hôte, Institut National de la Santé et de la Recherche Médicale and Université d’Auvergne, 63001 Clermont-Ferrand, France

**Keywords:** RNase E, RNA degradosome, Microdomain, MoRF, MoRE, SLiM, Multienzyme complex, Protein–protein interaction, mRNA degradation

## Abstract

RNase E of *Escherichia coli* is a membrane-associated endoribonuclease that has a major role in mRNA degradation. The enzyme has a large C-terminal noncatalytic region that is mostly intrinsically disordered (ID). Under standard growth conditions, RhlB, enolase and PNPase associate with the noncatalytic region to form the multienzyme RNA degradosome. To elucidate the origin and evolution of the RNA degradosome, we have identified and characterized orthologs of RNase E in the γ-Proteobacteria, a phylum of bacteria with diverse ecological niches and metabolic phenotypes and an ancient origin contemporary with the radiation of animals, plants and fungi. Intrinsic disorder, composition bias and tandem sequence repeats are conserved features of the noncatalytic region. Composition bias is bipartite with a catalytic domain proximal ANR-rich region and distal AEPV-rich region. Embedded in the noncatalytic region are microdomains (also known as MoRFs, MoREs or SLiMs), which are motifs that interact with protein and other ligands. Our results suggest that tandem repeat sequences are the progenitors of microdomains. We have identified 24 microdomains with phylogenetic signals that were acquired once with few losses. Microdomains involved in membrane association and RNA binding are universally conserved suggesting that they were present in ancestral RNase E. The RNA degradosome of *E. coli* arose in two steps with RhlB and PNPase acquisition early in a major subtree of the γ-Proteobacteria and enolase acquisition later. We propose a mechanism of microdomain acquisition and evolution and discuss implications of these results for the structure and function of the multienzyme RNA degradosome.

**Electronic supplementary material:**

The online version of this article (doi:10.1007/s00438-014-0959-5) contains supplementary material, which is available to authorized users.

## Introduction

Protein interaction networks have an important role in the organization of biological regulatory processes. Hubs proteins, which are network centers that associate with multiple partners, often contain intrinsically disordered (ID) protein, which lacks the propensity to form secondary and tertiary structure typical of globular proteins (Clarke et al. 2012; Tompa 2012; Oldfield and Dunker 2014). ID regions can interact with several protein partners at once and thereby accelerate interactions between partners. Although many protein interaction networks involving ID regions have been described in animals, plants, and fungi, research on the molecular basis of these interactions is recent and limited. In addition to integrative biophysical and molecular approaches, the elucidation of the molecular evolution of these networks should give clues to their origin and the selective pressure that shapes them. Here, we have analyzed the evolution of a hub protein conserved in the γ-Proteobacteria. RNase E is an essential endoribonuclease involved in general and regulated mRNA degradation in *Escherichia coli*. RNase E contains a large ID region encompassing elements involved in interactions with proteins and other ligands. Some of these elements have been identified as regions of increased structural propensity (RISPs) and, for this reason the term ‘microdomain’ was coined (Callaghan et al. 2004; Marcaida et al. 2006; Erce et al. 2009; Ait-Bara and Carpousis 2010). Comparable elements found in ID regions of eukaryotic hub proteins have been named molecular recognition features (MoRFs), molecular recognition elements (MoREs), or short linear motifs (SLiMs) (Mohan et al. 2007; Vacic et al. 2007; Tompa et al. 2014; Van Roey et al. 2014).

RNase E-like proteins with large N- and/or C-terminal noncatalytic regions are found in many bacteria and in the chloroplasts of some plants (Lee and Cohen 2003; Schein et al. 2008; Stoppel and Meurer 2012). RNase G of *E. coli* is a nonessential homolog of RNase E that has a high degree of conservation with the catalytic domain of RNase E (Li et al. 1999; Tock et al. 2000; Wachi et al. 1999). Together, RNase E and RNase G are the founders of the RNase E/G family of endoribonucleases (Kaberdin et al. 1998; Carpousis 2002; Condon and Putzer 2002). In *E. coli*, RNase E and RNase G are distinguished by the large noncatalytic C-terminal extension of RNase E, which is a region of ID protein over 500 residues in length (Callaghan et al. 2004). The noncatalytic region of RNase E is the scaffold for the assembly of the multienzyme RNA degradosome composed of RNase E, RhlB, enolase and PNPase (Carpousis 2007; Carpousis et al. 2009; Gorna et al. 2012; Bandyra et al. 2013). In other bacteria, RNase E homologs form degradosome-like complexes although composition is not conserved. Nevertheless, a common theme is the interaction of RNase E with exoribonucleases (PNPase or RNase R), RNA helicases (DEAD-box RNA helicases or Rho) and enzymes from central carbon metabolism (enolase or aconitase) (Jager et al. 2001; Lee and Cohen 2003; Jager et al. 2004; Purusharth et al. 2005; Hardwick et al. 2011).

**Fig. 1:**
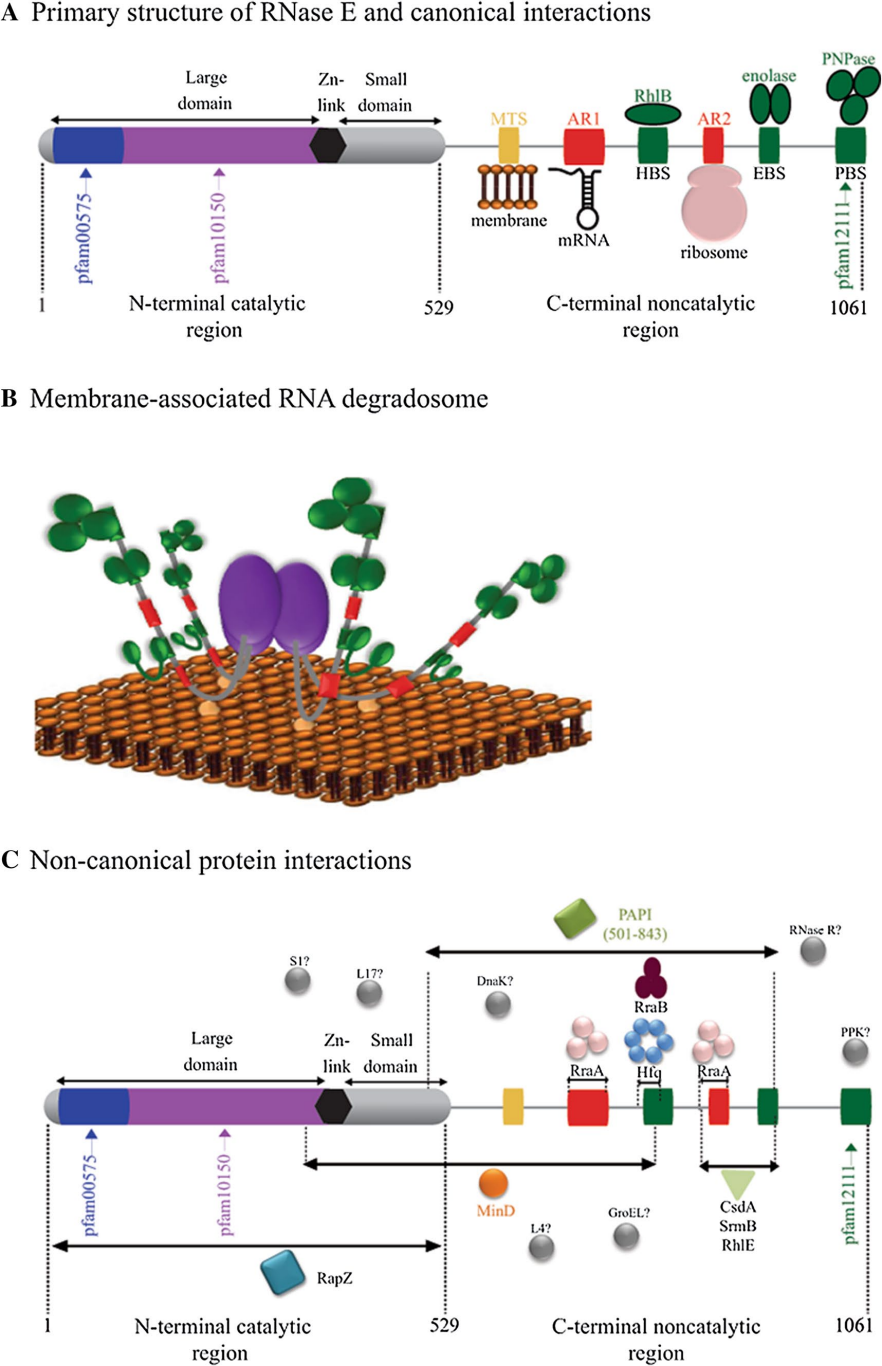
*E. coli* RNase E is a protein interaction hub. **a** Primary structure of *E. coli* RNase E showing the catalytic region (residues 1–529) and the noncatalytic region (residues 530–1,061 residues). The catalytic region is composed of a large domain, a zinc-link (Zn-link) and a small domain. The large domain is contains an S1 RNA binding motif (*blue* Pfam00575) and a metal-binding catalytic site (*purple*, Pfam10150). The RNase E tetrameric holoenzyme is a dimer of dimers. The large domain and Zn-link have a structural role in dimer formation; the small domain has a structural role in dimer and dimer–dimer interactions. The noncatalytic region (residues 530–1,061) contains microdomains responsible for the interaction between the RNase E and the inner plasma membrane (*yellow* MTS, membrane targeting sequence, residues 565–582), RNA (*red* AR1, arginine-rich 1, residues 604–644; AR2, arginine-rich 2, residues 796–814), and proteins to form the canonical RNA degradosome (*green* HBS, helicase binding site, residues 719–731; EBS, enolase binding site, residues 834–850 residues; PBS (Pfam12111), PNPase binding site, residues 1,021–1,061 residues). **b** *Cartoon* showing tetrameric RNase E holoenzyme bound to the inner cytoplasmic membrane and organization of the RNA degradosome. *Purple* catalytic core of RNase E; *gray* ID region; *yellow* MTS; *red* RNA binding sites; *green* protein binding sites and associated proteins. **c** Non-canonical protein interactions with *E. coli* RNase E. Hfq (residues 711–750) (Ikeda et al. 2011); CsdA, SrmB and RhlE (residues 791–843) (Khemici et al. 2004; Prud’homme-Genereux et al. 2004); RraA (residues 604–688 and 791–819) and RraB (residues 694–727) (Gao et al. 2006; Gorna et al. 2010); MinD (residues 378–724) (Taghbalout and Rothfield 2007); RapZ (residues 1–529) (Gopel et al. 2013); poly(A)polymerase (PAPI) (residues 501–843) (Raynal and Carpousis 1999; Carabetta et al. 2010). Other non-canonical interactions have been mentioned in the literature but the binding sites are unknown: RNase R (Carabetta et al. 2010); GroEL, DnaK, and polyphosphate kinase (PPK) (Miczak et al. 1996; Blum et al. 1997); ribosomal proteins such as S1, L4, L17 (Feng et al. 2001; Singh et al. 2009; Tsai et al. 2012) (color figure online)

RhlB, enolase and PNPase interact with *E. coli* RNase E via the helicase binding site (HBS), enolase binding site (EBS), and PNPase binding site (PBS), respectively (Fig. 1a). In the case of enolase and PNPase, the structure of these enzymes complexed with a polypeptide corresponding to the EBS or PBS has been solved by X-ray crystallography (Chandran and Luisi 2006; Nurmohamed et al. 2009, 2010). The HBS has been localized to a specific site by experimental work and by sequence comparison between RNase E homologs from *E. coli* and *Pseudoalteromonas haloplanktis* (Vanzo et al. 1998; Khemici and Carpousis 2004; Chandran et al. 2007; Worrall et al. 2008b; Ait-Bara and Carpousis 2010). The HBS, EBS and PBS interact with their protein partner with dissociation constants in the submicromolar range, which is sufficient to pull down the RNA degradosome from cell extracts and to reconstitute the complex from purified protein components (Miczak et al. 1996; Coburn et al. 1999). In addition to protein interactions, the noncatalytic region of RNase E contains elements involved in interactions with other ligands (Fig. 1a). AR1 and AR2 are RNA binding sites (Lopez et al. 1999; Leroy et al. 2002; Tsai et al. 2012). The membrane targeting sequence (MTS), which is located adjacent to the catalytic region, forms an amphipathic α-helix that anchors RNase E to the inner cytoplasmic membrane (Khemici et al. 2008). Since RNase E is a tetramer (Callaghan et al. 2004), the catalytic core is anchored to the inner cytoplasmic membrane with four ID regions that interact with proteins and RNA substrates extending into the cytoplasm (Fig. 1b).

In addition to the canonical RNA degradosome, a variety of RNase E-based complexes have been identified under non-standard conditions of growth (Fig. 1c). For example, RNase E, Hfq (an RNA binding protein) and SgrS (a small regulatory RNA), form a complex under conditions of phosphosugar stress that targets RNase E to the degradation of the *ptsG* mRNA (Morita et al. 2005; Worrall et al. 2008a). Other proteins that have been found associated with RNase E include RraA, RraB, CsdA, SrmB, RhlE, RNase R, MinD, RapZ, poly(A)polymerase, RNase R, GroEL, DnaK and several ribosomal proteins (see legend Fig. 1c for more detail). In some cases, these interactions involve conditions of stress in which the expression of a component such as SgrS is induced, suggesting that RNA degradosome remodeling might be driven by increasing the concentration of a non-canonical component. Plasticity in composition could be a general characteristic of a hub protein containing microdomains with moderate affinity for a protein partner.

Here, we have analyzed the molecular evolution of RNase E by taking advantage of an extensive set of complete genome sequences in the γ-Proteobacteria, which is a phylum of bacteria with diverse ecological niches and metabolic phenotypes that includes many important human, animal and plant pathogens (Gao et al. 2009; Williams et al. 2010). The last common ancestor (LCA) of the γ-Proteobacteria dates to at least 500 million years ago and the evolutionary distance between orders in this phylum is as large as between animals, plants and fungi. We show that RNase E and RNase G form distinct orthologous groups that have been inherited vertically. Intrinsic disorder, composition bias and tandem sequence repeats are conserved features of the noncatalytic region of RNase E. We propose a mechanism for acquisition and evolution of microdomains based on these results. Using a methodology that detects conserved sequence motifs, we have elucidated the evolutionary history of microdomains in the noncatalytic region of RNase E. We infer that ancestral RNase E had a large ID noncatalytic region and microdomains involved in membrane association and RNA binding. The membrane-associated RNA degradosome is, therefore, a hallmark of the γ-Proteobacteria.

## Materials and methods

Data from completely sequenced bacterial genomes were retrieved from a database (CGDB) maintained in our laboratory. CGDB includes information automatically retrieved from EMBL files and results of local sequence analysis such as the annotation of conserved domains and homology links between sequences. At the time of the analysis the database included 1,053 genome sequences from 679 species. Conserved domains (CD) were retrieved from the NCBI (Marchler-Bauer et al. 2011). We used the RPSBLAST annotation of COG1530, COG0148 and COG1185 to retrieve RNase E/G, enolase and PNPase homologs from γ-Proteobacteria complete genomes. The RNase R sequences were identified with the Pfam00773 domain. RPSBLAST was used to predict CDs (Altschul et al. 1997) and we retained only the best non-overlapping hit(s). The homology links were computed at the protein level with BLASTP. We compared all proteins of each pair of genomes. The results were then parsed to annotate each pair of proteins as best hit, ortholog or one-to-one ortholog (Fitch 2000). If protein *a* from genome *A* is the best hit of protein *b* from genome *B* and if *b* is the best hit of *a*, then (*a*, *b*) are orthologs; otherwise (*a*, *b*) are best hits. With this definition of orthology, we cannot exclude that at least in one genome one or more duplications occurred after the divergence of both species (species paralogs). This is detectable at the level of the sequence if the blast score between putative paralogs in one genome is greater than the blast score between *a* and *b*. In the absence of species paralogs, (*a*, *b*) are one-to-one orthologs. With this more restrictive definition (one-to-one orthology), proteins *a* and *b* have a greater chance to have a conserved function (Fitch 2000).

### Protein families and subfamilies

The structure in families/subfamilies of the RNase E/G proteins was explored from the homology links computed between pairs of proteins. We built an unweighted and undirected graph from the list of one-to-one orthologous protein pairs. This graph is composed of highly dense regions (groups) that are loosely connected. In general, groups were composed of proteins from different organisms with little or no paralogy. The groups define subfamilies of proteins that have a high probability to have conserved the same or very similar function(s) in the different organisms. We used a Markov Cluster Algorithm (Van Dongen and Lens 2000) to extract communities from the graph. The granularity (number of groups) was controlled by the inflate factor.

### Alignment and phylogeny

Alignments were done with MUSCLE (Edgar 2004) with the default parameters and edited with Jalview (http://www.jalview.org) (Waterhouse et al. 2009) to extract the common core of conserved residues. We used trimAl (Capella-Gutierrez et al. 2009) to eliminate poorly aligned positions (Alba et al. 2002) and divergent regions from the multiple alignments. The phylogenetic trees were computed with PhyML (Guindon and Gascuel 2003). We used ProtTest (Abascal et al. 2005) to select the optimal combination of parameters. The most frequent combination was the LG model of sequence evolution with the Γ-correction (four categories of evolutionary rates), shape parameter and proportion of invariant sites estimated from the data. Replicates (100) were done for the nonparametric bootstrap analysis. The trees were drawn and annotated with the Interactive Tree Of Life web server (iTOL, http://itol.embl.de/index.shtm) (Letunic and Bork 2007). The trees were used to validate and display evolutionary relationships of the families/subfamilies obtained with MCL on the full length proteins.

### Species tree of the γ-Proteobacteria

We selected a sample of COG families according to their conservation in γ-Proteobacteria genomes. The alignments for each selected COG family were created using MUSCLE with the default parameters. TrimAl was used to edit alignments and to discard COG families which did not exhibit a high quality alignment. The aligned sequences of 83 COG families for the 152 species were concatenated together to produce a single alignment of 26,581 positions. When a species did not have a record for a COG family, the missing sequence was replaced by gaps. The species tree was inferred as described above with PhyML according to the ProtTest results obtained with each alignment. The same concatenated alignment was also used to infer 100 nonparametric bootstrap replicates. During this procedure, we observed that a few strains with very fast evolutionary rates impact the quality of the tree. *Candidatus Blochmannia floridanus*, *Blochmannia pennsylvanicus*, *Blochmannia vafer*, *Hamiltonella defense*, *Moranella endobia*, *Riesia pediculicola*, *Baumannia cicadellinicola*, *Carsonella ruddii* and *Wigglesworthia glossinidia* are endosymbionts with small A + T-rich genomes. It has been proposed that their misplacement on the tree is due to compositional attraction (Williams et al. 2010). The tree that we obtained is in good agreement with recently published work (Gao et al. 2009; Williams et al. 2010). The *Enterobacteriales* arose from the VAAP clade (*Vibrionales*, *Aeromonadales*, *Alteromonadales*, *Pasteurellales*). Another subdivision includes the PO clade (*Pseudomonadales* and *Oceanospirillales*). The most anciently diverging linages in the γ-Proteobacteria are found in deeper branches. As reported previously (Williams et al. 2010), species of three orders (*Alteromonadales*, *Pseudomonadales*, *Oceanospirillales*) are not monophyletic.

### Intrinsically disordered protein, compositional biases and motif identification

Intrinsically disordered (ID) segments in RNase E homologs were detected by DISOPRED2 (Ward et al. 2004). Compositional biases (CB) in amino acid content was analyzed with the LPS-annotate WEB server (Harbi et al. 2011) using default parameters. The annotation of tandem repeats in protein sequences was done with XSTREAM (Newman and Cooper 2007). The identification of the conserved CPxCxGxG motif corresponding to the Zn-link was achieved with scan-for-matches program (Dsouza et al. 1997). The search for conserved motifs was done using MEME (http://meme.nbcr.net/meme) (Bailey et al. 2006, 2009). In this analysis, we retained only one strain per species. We used Jalview (Clamp et al. 2004) to remove the N-terminal conserved catalytic domain and removed all gaps from C-terminal regions. The unaligned sequences were submitted to MEME with a motif width between 10 and 25 amino acids. We searched for a maximum of 30 motifs with an occurrence of zero or one motif per sequence since we did not expect that each motif would be present in all sequences from the training set. With these parameters we reduced the chance of missing the identification of biologically relevant motifs. The results of MEME were used by MAST to annotate the motifs.

The first occurrence of a motif during the evolution of the γ-Proteobacteria was inferred using the ancestral character estimation (ace) function of the ape R package (Paradis et al. 2004). We used the maximum likelihood estimation and a model with two character states and unequal transition rates. The presence (1) or absence (0) of the motif at each tree leaf was encoded in a numeric matrix. With this method, the probability of the presence or absence of the motif at each ancestral node was inferred on the species tree.

## Results

### Relationship of RNase E and RNase G

**Fig. 2:**
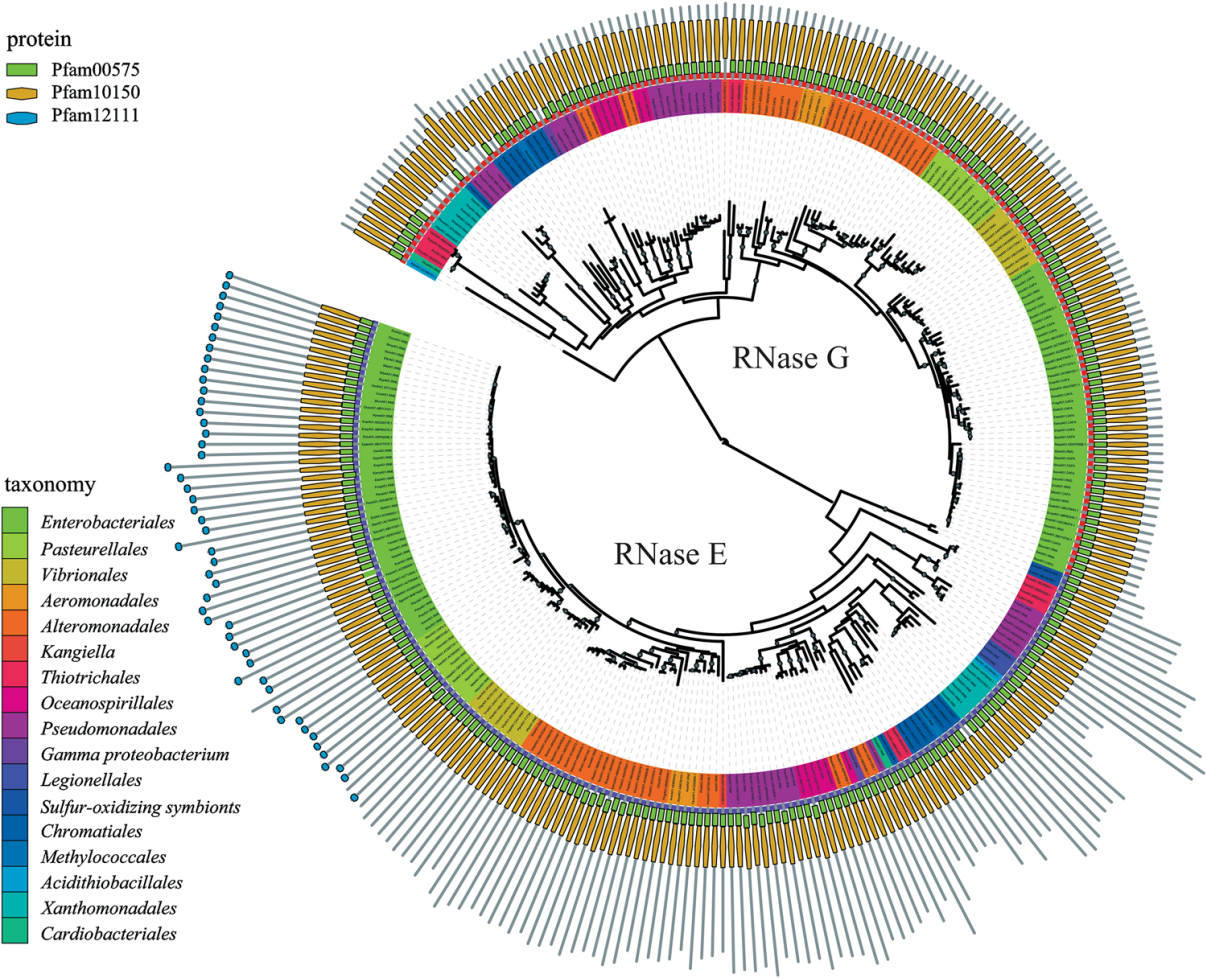
RNase E and RNase G form distinct orthologous groups in the γ-Proteobacteria. Phylogenetic trees of RNase E and RNase G homologs were constructed as described (“Materials and methods”). *Gray dots* indicate branches with high bootstrap support. *Inner circle* tree leaves colored according to the taxonomy (taxonomy key). *Center circle* RNase G, *red*; RNase E, *purple*. *Outer circle* *line diagram* of primary structure of RNase E and RNase G homologs showing the conserved Pfam domains (protein key) (color figure online)

To better understand the relationship of RNase E and RNase G, we used the COG1530 profile from the CD database (NCBI) with RPSBLAST to obtain a high quality annotation of the RNase E and RNase G homologs in the γ-Proteobacteria. We used one-to-one orthology links computed between pairs of full length proteins to classify the sequences into families by the MCL method. We obtained a phylogenetic tree with two orthologous groups corresponding to RNase E and RNase G demonstrating that these homologs can be unambiguously discriminated (Fig. 2). The RNase E protein sequence tree is concordant with the species tree showing that RNase E was inherited vertically. The RNase G protein sequence tree is concordant with a large subtree, which is comprised of the VAAP clade (*Vibrionales*, *Aeromonadales*, *Alteromonadales and Pasteurellales*) and *Enterobacteriales*. The phylogeny suggests that the ancestral γ-Proteobacteria encoded homologs of RNase E and RNase G. These results show that RNase E and RNase G in the γ-Proteobacteria form well-separated orthologous groups that have an ancient origin predating the radiation of the γ-Proteobacteria. Although nonessential in *E. coli*, these results suggest that RNase G has an important conserved function that is distinct from RNase E.

### Distribution of RNA degradosome components

**Fig. 3:**
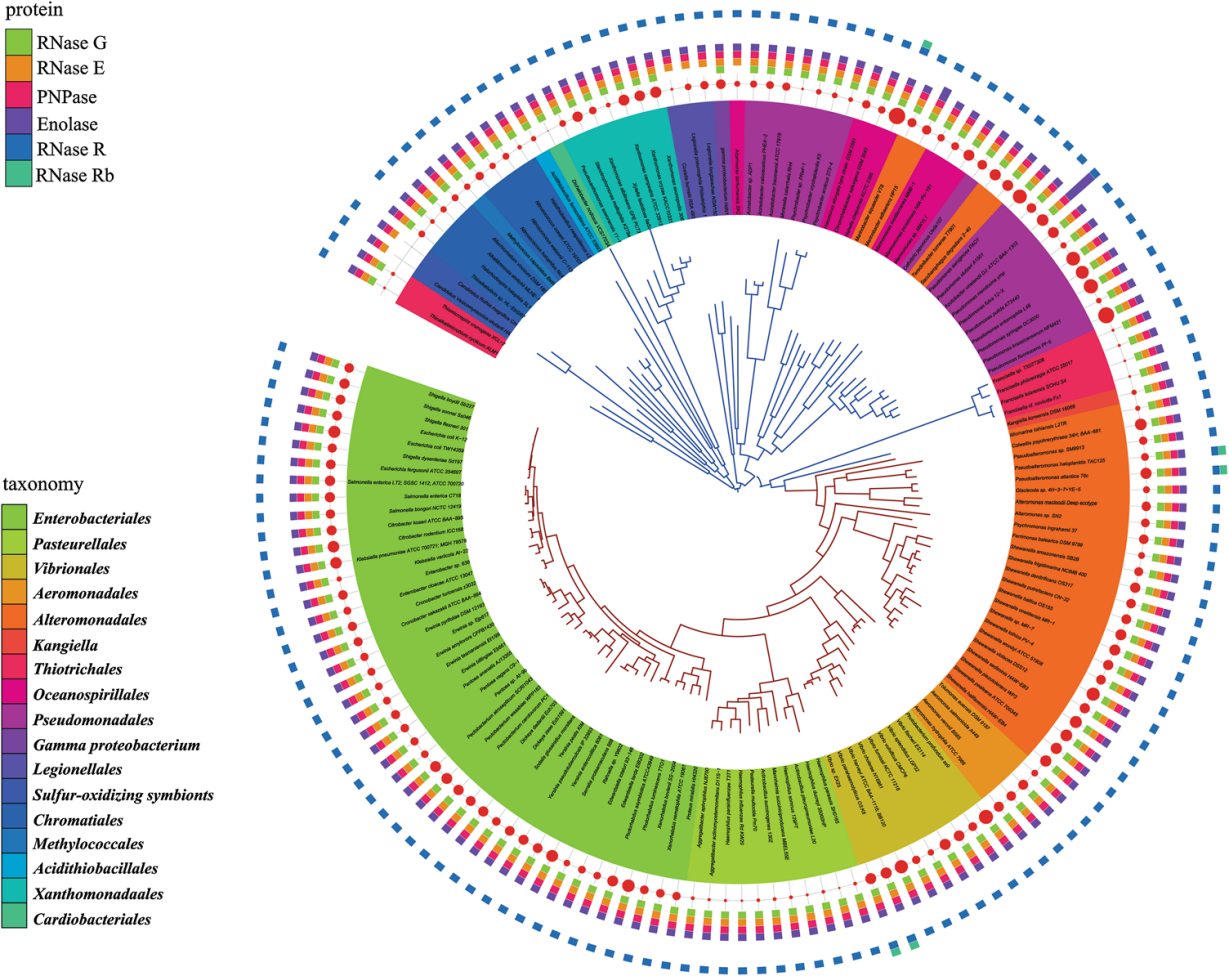
Distribution of RNase G, RNase E, PNPase, enolase and RNase R. The phylogenetic tree of γ-Proteobacteria species was constructed as described (“Materials and methods”). The *blue* branches correspond to a subdivision that includes the PO clade (*Pseudomonadales* and *Oceanospirillales*); the *red* branches to the VAAP clade (*Vibrionales, Aeromonadales, Alteromonadales, Pasteurellales*) and *Enterobacteriales*. *Inner circle* tree leaves according to the taxonomy (taxonomy key). *Center circle* *red dots* indicate genome size. *Outer circle* distribution of proteins (protein key). The gene encoding enolase is present in multiple copies in *Marinobacter adhaerens* and *Azotobacter vinelandii*. A second copy of the gene encoding RNase R is present in a few species and the protein is labeled RNase Rb (color figure online)

Previous work has demonstrated that RhlB, enolase and PNPase interact with RNase E to form the RNA degradosome in *E. coli* and closely related species of bacteria. In *P. haloplanktis* (*Alteromonadales*), an RNA degradosome-containing RNase E, RhlB and PNPase, but lacking enolase has been characterized (Ait-Bara and Carpousis 2010) whereas in the *Pseudomonas syringae* Lz4W (*Pseudomonadales*) an RNA degradosome composed of RNase E, the DEAD-box helicase RhlE and RNase R has been identified (Purusharth et al. 2005). We were, therefore, interested in the distribution of orthologs of RNA degradosome components in the γ-Proteobacteria and we included RNase G in this analysis. RhlB and RhlE are well conserved and widely distributed in the γ-Proteobacteria (Lopez-Ramirez et al. 2011) and both are encoded in the genomes of *E. coli* and *P. syringae* Lz4W. Figure 3 shows the distribution of RNase G, RNase E, PNPase, enolase and RNase R mapped onto the species tree of the γ-Proteobacteria. In a limited number of bacteria, there are two genes encoding RNase R. The enzyme encoded by the additional gene is annotated as RNase Rb. Since some of the bacterial species in Fig. 3 are endosymbionts that have undergone genome shrinkage, we have indicated genome size by a red dot. The main conclusion of this analysis is that RNase E, PNPase, enolase and RNase R are ubiquitous in the γ-Proteobacteria, even in species that have undergone significant genome shrinkage. RNase G with one exception is also widespread; it has been lost in the *Legionellales*. These results show that RNase G and the components of the RNA degradosome are omnipresent in the γ-Proteobacteria. We, therefore, conclude that differences in RNA degradosome composition cannot be explained by the presence or absence of a degradosome component.

### Conserved features of the noncatalytic region of RNase E

**Fig. 4:**
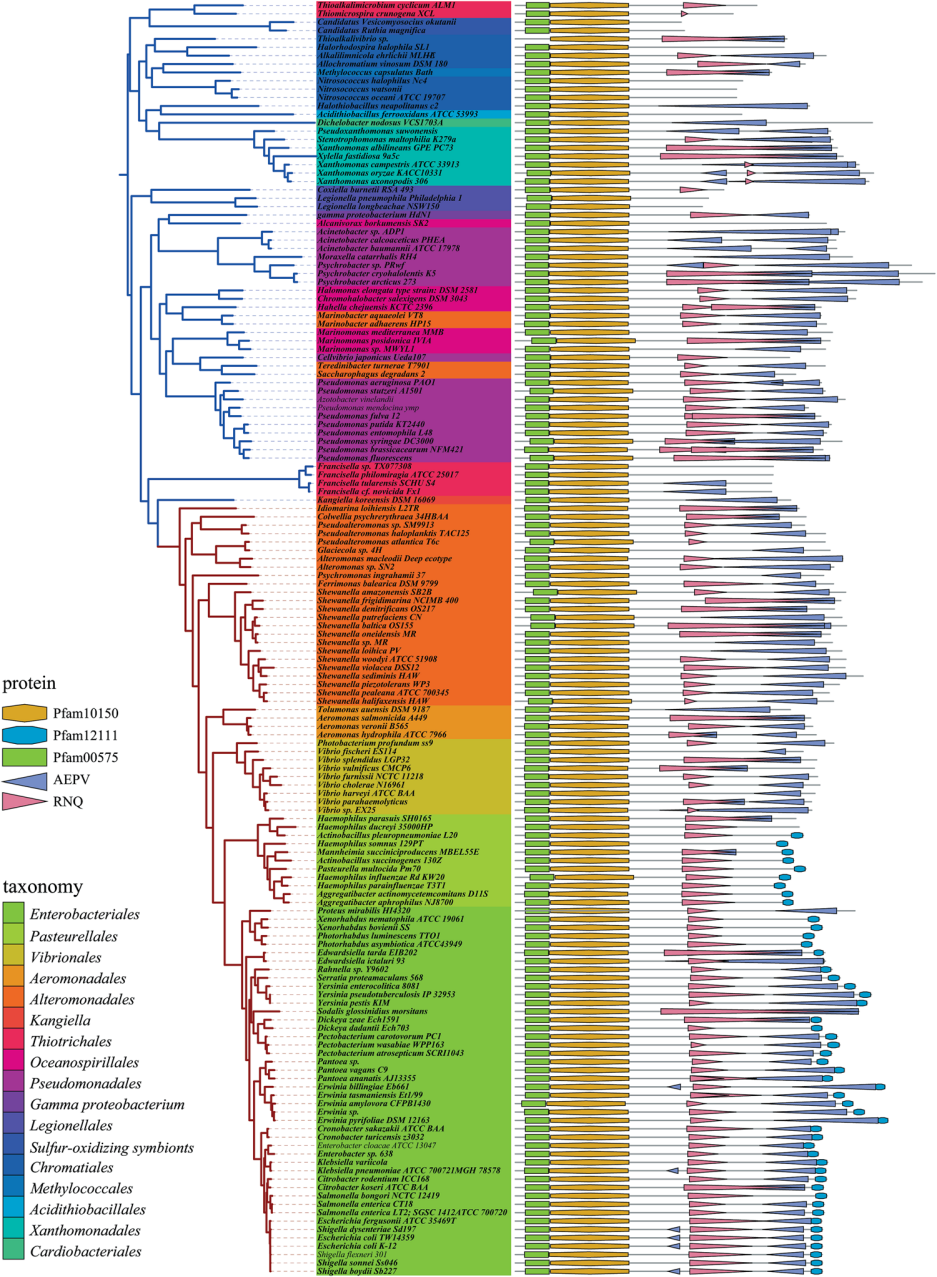
Composition bias in the noncatalytic region of RNase E orthologs. Primary structure of a representative selection of RNase E homologs (*right half of panel*) is mapped to the species tree of the γ-Proteobacteria (*left half of panel*), which was constructed as described (“Materials and methods”). The *blue* branches correspond to a subtree that includes the PO clade (*Pseudomonadales* and *Oceanospirillales*); the *red* branches to the VAAP clade (*Vibrionales, Aeromonadales, Alteromonadales, Pasteurellales*) and *Enterobacteriales*. Tree leaves are *color* coded according to taxonomy (key). *Symbols* for Pfam domains and composition bias (CB) are indicated in the protein key. Note that the *symbols* for CB represent the region of bias, they do not imply a gradient or directionality (color figure online)

Computational analyses as well as biochemical and biophysical studies have shown that the noncatalytic region of RNase E in *E. coli*, *Vibrio Angustum* S14 and *P. haloplankti*s TAC125 is mostly intrinsically disordered (Callaghan et al. 2004; Erce et al. 2009; Ait-Bara and Carpousis 2010). Analysis of the complete set of *E. coli* K12 proteins using DISOPRED2 (Ward et al. 2004) shows that of approximately 50 very large proteins (greater than 1,000 residues), RNase E, FtsK and MukB have extensive ID regions (greater than 50 %) (Fig. S1). Using DISOPRED2, nearly all RNase E orthologs in the γ-Proteobacteria are predicted to have a large ID region, which varies in length from less than 500 to greater than 800 residues (Fig. S2A). ID regions are often associated with composition bias (CB) in amino acid residues (Tompa 2003; Simon and Hancock 2009). We searched for CB with the LPS-annotate WEB server (Harbi et al. 2011). When we filtered with a stringent *P* value (1.0 e^−15^), CB was restricted to the noncatalytic region (Fig. S2B). CB can be clustered into two groups (Fig. S3A): one with a high frequency of arginine (R), asparagine (N) and glutamine (Q) residues (0.214, 0.101 and 0.091, respectively); the other with a high frequency of alanine (A), glutamate (E), proline (P) and valine (V) residues (0.208, 0.138, 0.106 and 0.127, respectively) (Fig. S3B). In contrast, tryptophan (W), phenylalanine (F), tyrosine (Y), cysteine (C), leucine (L), methionine (M), and histidine (H) are underrepresented. In Fig. 4, the left half of the panel shows the species tree and the right half of the panel shows the corresponding RNase E homologs represented as a line diagram corresponding to the primary sequence. The noncatalytic region in a majority of RNase E homologs is composed of an RNQ-rich region (pink) followed by an AEPV-rich region (blue). These regions have a tendency to contain repeated sequences such as REE and AEVP. Using XSTREAM (Newman and Cooper 2007), we observed a high frequency of tandem repeat sequences in the noncatalytic region of RNase E homologs (Table S1 and Fig. S2C). In conclusion, these results show that intrinsic disorder, composition bias and tandem repeat sequences are conserved features of the noncatalytic region of RNase E.

### Conserved sequence motifs in the noncatalytic region of RNase E

**Fig. 5:**
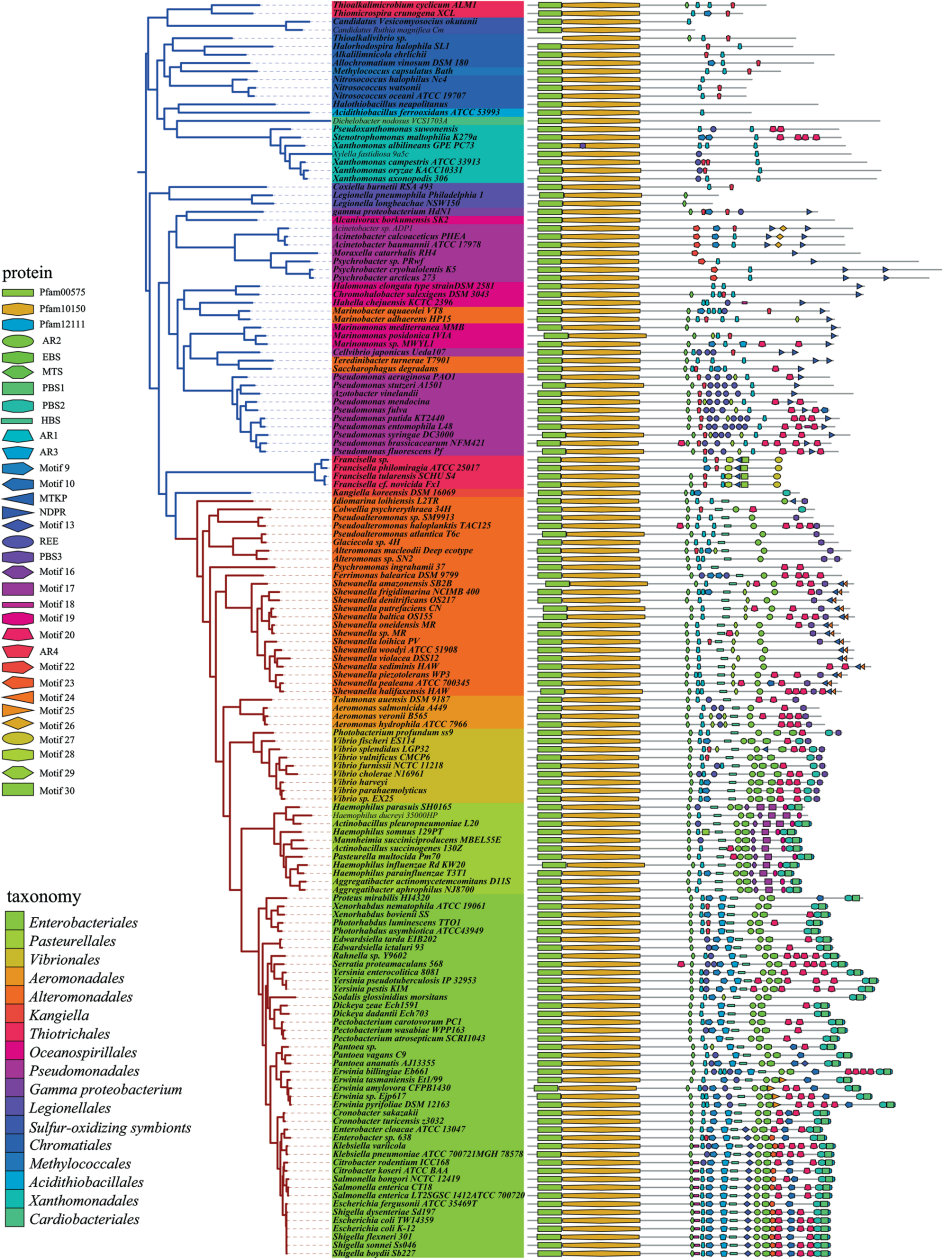
Conserved sequence motifs in the noncatalytic region of RNase E orthologs. The primary structure of a representative selection of RNase E homologs (*right half of panel*) is mapped to the species tree of the γ-Proteobacteria (*left half of panel*), which was constructed as described (“Materials and methods”). The *blue* branches correspond to a subdivision that includes the PO clade (*Pseudomonadales* and *Oceanospirillales*); the *red* branches to the VAAP clade (*Vibrionales, Aeromonadales, Alteromonadales, Pasteurellales*) and *Enterobacteriales*. Tree leaves are *color* coded according to taxonomy (key). *Symbols* for Pfam domains and conserved sequence motifs are indicated in the protein key (color figure online)

We searched for conserved sequence motifs in the noncatalytic region of RNase E orthologs using MEME, which is a program that discovers motifs by searching a library of protein sequences (Bailey et al. 2006, 2009). We analyzed a complete sample of RNase E homologs from the γ-Proteobacteria using sequences in which the catalytic core was deleted. Thirty motifs were ranked according to P value (Table 1). MEME detected all microdomains of the *E. coli* RNase E homolog that have been previously identified and characterized experimentally. Motifs 7, 1, and 3 correspond to the AR1, AR2 and MTS, respectively. With respect to the protein binding sites in *E. coli* RNase E, motifs 5, 2 and 4 correspond to the HBS, EBS, and PBS, respectively. Figure 5 is a snapshot of the distribution of conserved sequence motifs in the noncatalytic region of RNase E orthologs in the γ-Proteobacteria. In the few cases where an MTS was not detected by MEME, inspection of the region where the MTS is normally located showed that these homologs have an amphipathic α-helix that could serve as an MTS. Inspection of the figure reveals that evolution of the motifs is concordant with the species tree. We inferred the ancestral state of each motif on each node of the γ-Proteobacteria species tree (Table 1 and Fig. S4). Twenty-four motifs are predicted with high probability (*P* ≥ 0.92) to have been acquired once; one motif (11) is predicted to have been acquired independently in two clades. Five motifs (9, 14, 20, 21 and 29) corresponding to low complexity sequences enriched in arginine residues (R) or alanine, proline, aspartic acid and valine residues (AEPV) are predicted to have been acquired multiple times. The MTS and AR1 are inferred to have been present in the ancestral RNase E of the γ-Proteobacteria. Following the branch leading to the *Enterobacteriales*, we observe acquisition of AR2, PBS3 and HBS in the LCA of the VAAP clade; acquisition of EBS and PBS2 in the LCA of *Vibrionales, Pasteurellales* and *Enterobacteriales*; acquisition of PBS1 and the loss of PBS3 in the LCA of *Pasteurellales* and *Enterobacteriales*; acquisition of AR3 in the LCA of *Enterobacteriales*. Motifs 10, 13, 18 and 23 appear successively along the branch leading to *E. coli*. Other motifs appear in the *Enterobacteriales* (motif 25 in *Erwinia*) and in the VAAP clade (motifs 16, 17 and 19 in *Pasteurellales*; motifs 11 and 24 in *Shewanella*). Motif 12 appears in the LCA of the PO clade (*Pseudomonadales* and *Oceanspirillales*) and then motif 22 in *Acinetobacter* and *Psychrobacter* and motif 26 in *Acinetobacter*. In addition to identification of known microdomains, we have mapped conserved sequences in the *Enterobacteriales*, including motifs 8, 10, 13, 18, and 23, which are candidates for sites of interaction with non-canonical proteins such as Hfq and poly(A)polymerase (Fig. 1c). Motif 12, which is restricted to the PO clade, is a candidate for an interaction with RNase R or RhlE, which have been shown to associate with the RNase E in *Pseudomonas syringae* (Purusharth et al. 2005). In the *Methylococcales*, *Acidithiobacillales* and *Xanthomonadales*, the RNase E orthologs are highly divergent and the only conserved features are the AR1, MTS and compositionally biased motifs (AEVP-rich, REE, and AR4). In conclusion, these results show that it is possible to map conserved sequence motifs that correspond to known and putative microdomains and to infer their evolutionary history.

**Table 1:** MEME-discovered motifs in the noncatalytic region of RNaseE homologs (color table online)

Rank and motif		Length	Name	Taxonomic distribution	Gain^a^
1		21	AR2	*Aeromonadales, Alteromonadales, Vibrionales, Pasteurellales, Enterobacteriales*	0.99
2		25	EBS	*Vibrionales, Pasteurellales, Enterobacteriales*	0.99
3		14	MTS	Almost all γ-Proteobacteria	0.92
4		25	PBS1	*Pasteurellales, Enterobacteriales*	0.98
5		25	HBS	*Aeromonadales, Alteromonadales, Vibrionales, Pasteurellales, Enterobacteriales*	1.00
6		25	PBS2	*Vibrionales, Pasteurellales, Enterobacteriales*	0.95
7		14	AR1 RNQ-rich	γ-Proteobacteria	1.00
8		25	AR3 RNQ-rich	*Enterobacteriales*	1.00
9*		25	RNQ-rich	*Enterobacteriales* and less conserved in a few other species	Multiple
10		25	AEPV-rich	*Escherichia, Klebsiella, Salmonella, Shigella, Enterobacter, Citrobacter, Erwinia, Pantoea*	0.99
11		19	MTKP	*Shewanella, Thiotrichales*	1.00
					1.00
12		19	NDPR	*Oceanospirillales, Pseudomonadales*	1.00
13		25	AEPV- rich	*Escherichia, Klebsiella, Salmonella, Shigella, Enterobacter, Citrobacter*	1.00
14*		19	REE	*Pseudomonadales, Xanthomonadales* and less conserved in other species	Multiple
15		19	PBS3	*Alteromonadales, Aeromonadales, Vibrionales*	0.92
16		17		*Pasteurellales*	1.00
17		25		*Pasteurellales*	1.00
18		19		*Escherichia, Klebsiella, Salmonella, Shigella, Enterobacter, Citrobacter*	1.00
19		14		*Pasteurellales*	1.00
20		25	AEPV-rich	Largely distributed with low p value and repeats	Multiple
21*		14	AR4 RNQ-rich	*Pseudomonadales* and other species with low p value	Multiple
22		23		*Acinetobacter, Psychrobacter*	0.99
23		19		*Escherichia, Klebsiella, Salmonella, Shigella, Enterobacter, Citrobacter*	0.99
24		13		*Shewanella*	1.00
25		25		*Erwinia*	1.00
26		25		*Acinetobacter*	1.00
27		25		*Francisella*	1.00
28		25		*Francisella*	1.00
29*		14		*Alteromonadales, Pseudomonadales*	Multiple
30		25		*Francisella*	1.00

^a^Twenty-four motifs are predicted to be acquired once as measured by the ace function of the ape R package (scaled likelihood of gain). Six motifs, including four arginine-rich motifs (*), are predicted to have been acquired more than once

## Discussion

### Microdomain identification

Here, we have identified microdomains by searching for conserved sequence motifs in the noncatalytic region of a large sample of RNase E orthologs and analyzing their inheritance during the evolution of the γ-Proteobacteria. Some but not all of these motifs have been identified as regions of increased structural propensity (RISPs), which are local sites of limited residual structure that nucleate a disorder-to-order transition upon interaction with a structured partner (Lee et al. 2000; Fuxreiter et al. 2004). Microdomains that give a clear RISP signal include the MTS, EBS and PBS (Callaghan et al. 2004; Erce et al. 2009; Ait-Bara and Carpousis 2010). The molecular basis for the detection of the RISPs involves the propensity to form secondary structures such as α-helices or β-sheets. For example, the MTS forms an amphipathic α-helix that is stabilized by interaction with phospholipid bilayers (Khemici et al. 2008). However, the AR1, AR2 and HBS do not have a detectable RISP signal even though there is substantial experimental evidence for interactions with these microdomains. For this reason, we suggest that the phylogenomic approach employed here is more inclusive than methods based on predicting disorder/order transitions. The only caveat is that the evolutionary depth of the sample of sequences needs to be sufficiently large to assure statistically meaningful results. Although we have used the term microdomain to be consistent with previous work on RNase E, the sequences identified here can be regarded as SLiMs since their identification does not rely on the detection of RISPs.

### Microdomain evolution

RNA degradosome components are conserved throughout the γ-Proteobacteria. Change in RNA degradosome composition is, therefore, driven by evolution of RNase E microdomains, which occurs in the presence of interacting partners. Analysis of conserved sequence motifs in the noncatalytic region of RNase E orthologs of the γ-Proteobacteria has permitted reconstruction of the history of microdomain evolution. Our results show that the EBS was acquired in the ancestor of *Vibrionales*, *Pasteurellales* and *Enterobacteriales* either as a duplicated site that was subsequently reduced to single copy in the *Pasteurellales* and *Enterobacteriales* or as a single site that was duplicated in the *Vibrionales*. The HBS and PBS were acquired together at the branch of the species tree where the VAAP clade emerged, which is consistent with known functional interactions between RNase E, RhlB and PNPase in *E. coli*. The physical assembly of the RNA degradosome is necessary for the cooperation of the RNA helicase activity of RhlB with the ribonuclease activities of RNase E and PNPase (Coburn et al. 1999; Khemici and Carpousis 2004; Khemici et al. 2005). This functionality is very likely the selective pressure for conserving the RNase E–RhlB–PNPase partnership in a large subtree of the γ-Proteobacteria. Whereas the HBS is highly conserved, the acquisition and evolution of the PNPase binding site is complex. PBS1 (motif 4) and PBS2 (motif 6) are present in RNase E orthologs of the *Pasteurellales* and *Enterobacteriales* (Table 1). In the *Vibrionales*, these motifs are replaced by PBS3 (motif 15), which also occurs frequently in the *Aeromonadales* and *Alteromonadales* where it has been shown to be a PNPase binding site in the RNase E ortholog of *P. haloplanktis* (Ait-Bara and Carpousis 2010). In *Shewanella*, two other motifs are present at a similar position: motifs 11 and 24. Remarkably, motif 11 also shares sequence similarities with PBS2 and PBS3. Localization at the C-terminal end of RNase E and sequence similarities suggest that all these motifs could have been derived from a common ancestral microdomain.

### Constraints on microdomain evolution

Experimental work has shown that the RNase E–PNPase interaction in *P. haloplanktis* and *E. coli* is species specific (Ait-Bara and Carpousis 2010). The interaction in *E. coli* involves the extension of a β-sheet on the surface of PNPase (Nurmohamed et al. 2009). We propose that the ancestral PBS microdomain evolved to conserve the RNase E–PNPase interaction during speciation and that the sequence of the microdomain co-varied with changes on the interaction surface of PNPase, for example by neutral substitutions that conserve β-sheet structure. In contrast, *P. haloplanktis* RNase E interacts with *E. coli* RhlB and the sequence of the HBS is conserved over a large evolutionary distance (VAAP clade and *Enterobacteriales*). Since *E. coli* and *P. haloplanktis* RhlB are highly conserved (same length, 65 % sequence identity), we propose that HBS conservation is due to constraints involved in the allosteric control of RhlB activity by its interaction with RNase E (Vanzo et al. 1998; Chandran et al. 2007; Worrall et al. 2008b; Ait-Bara and Carpousis 2010). These considerations lead to the conclusion that microdomain differentiation will depend on the functionality of the interaction. If the interaction involves a simple protein interface, then the sequence of the microdomain can vary with neutral substitutions that maintain contacts at the interface. The drift in sequence and structure can lead to species-specific interactions. On the other hand, interactions involving constraints such as the allosteric control of activity could lead to the conservation of sequence and structure over a large evolutionary distance. The MTS and AR1 are additional examples of highly conserved microdomains. Conservation of their sequence and structure likely involve constraints on specificity and affinity for their ligands (phospholipid membrane and RNA, respectively).

### Mechanism of microdomain acquisition and evolution

Large regions of ID protein have unusual properties such as extended conformation and spring-like characteristics that could have a role in functional interactions between components of the RNA degradosome and binding and release of RNA substrates (Tompa 2003). Composition bias is a hallmark of ID protein. One possible role of the RNQ- and AEPV-sequences is that composition affects physical properties such as charge, hydrophobicity, flexibility and compaction. These considerations lead us to suggest that the functional properties of ID protein are the underlying selection pressure for the conservation of RNase E orthologs with large noncatalytic regions. We propose that microdomain acquisition and evolution is a consequence of processes that generate and maintain large ID regions.

Our snapshot of the primary structure of extant RNase E homologs in the γ-Proteobacteria suggests that the expansion of tandem amino acid repeats in ID protein has continued throughout the evolution of the γ-Proteobacteria. The variability of the length of the noncatalytic region from less than 500 residues to more than 800 residues is consistent with a stochastic process in which expansion is balanced by deletion. Regions biased in composition have a tendency to be arranged as tandem amino acid repeats (Table S1), which result in direct repeats in the coding sequence. These regions can expand by DNA replication slippage. They would also be subject to deletion between direct repeats. With the accumulation of point mutations, expansion and deletion would decrease in frequency and some segments would become fixed if they acquired a new function such as an interaction with protein or another ligand (conversion of repeat sequence to microdomain). Such evolutionary processes affect the length, composition and organization of domains and have been suggested to reorganize protein interaction networks during evolution (Dosztanyi et al. 2006). Maintenance of a large ID region by the expansion of tandem amino acid repeats, offset by deletion and fixation by point mutation, provides a plausible mechanism for the acquisition and evolution of microdomains.

## Conclusions

Here we have focused on the γ-Proteobacteria because of the availability of large sample of complete genome sequences and because experimental work with *E. coli*, *P. haloplanktis*, *V. angustum* and *P. syringae* has permitted interpretation of microdomain evolution in terms of interactions with known proteins and ligands (Purusharth et al. 2005; Marcaida et al. 2006; Erce et al. 2009; Ait-Bara and Carpousis 2010). We can nevertheless draw conclusions about the evolution of RNase E in other phyla. An unanticipated result of this work is that RNase E and RNase G form orthologous groups. This is likely to be true in other phyla where RNase E and RNase G are encoded as paralogs since our results show that these ribonucleases differentiated before the emergence of the γ-Proteobacteria. When and how RNase E and RNase G differentiated is an open question. An RNA degradosome containing RNase E, RhlB and PNPase is restricted to a large subtree of the γ-Proteobacteria. The microdomains in the noncatalytic region that are responsible for the interaction with RhlB and PNPase are characteristic of RNase E orthologs in this subtree. Nevertheless, RNase E interactions with exoribonucleases and RNA helicases are widespread (Lee and Cohen 2003; Jager et al. 2004; Purusharth et al. 2005; Hardwick et al. 2011). RNA degradosomes in other phyla of bacteria with compositions similar to the *E. coli* multienzyme complex are, therefore, likely to have arisen several times independently. The recent demonstration of a PNPase interaction involving a conserved microdomain in RNase E orthologs of Cyanobacteria is a clear example of independent acquisition of a PBS in a phylum of bacteria that is distantly related to the Proteobacteria (Zhang et al. 2014). We have proposed a mechanism of microdomain acquisition and evolution that could lead to the ‘capture’ of enzymes that cooperate with RNase E in the degradation of mRNA. We speculate that the formation of RNase E-based multienzyme complexes adds a level of regulation in the coordination and control of mRNA degrading enzymes that is advantageous for the organism. Finally, the MTS is restricted to RNase E homologs of the β- and γ-Proteobacteria (Khemici et al. 2008). An amphipathic α-helix is not detected at a similar position in homologs from the α-Proteobacteria and RNase E has been reported to be associated with the nucleoid in *Caulobacter crescentus* (Llopis et al. 2010). Whether RNase E is generally membrane associated is an open question. It is nevertheless interesting that RNase Y, a ribonuclease in *Bacillus subtilis* involved in mRNA degradation with activities similar to RNase E, associates with the inner cytoplasmic membrane by an N-terminal transmembrane domain (Shahbabian et al. 2009). Membrane association of mRNA degrading enzymes could, therefore, be widespread in bacteria.

## Electronic supplementary material

Below is the link to the electronic supplementary material.

Supplementary material 1 (TIFF 1047 kb)

Supplementary material 2 (TIFF 2837 kb)

Supplementary material 3 (TIFF 3259 kb)

Supplementary material 4 (TIFF 2772 kb)

Supplementary material 5 (TIFF 1154 kb)

Supplementary material 6 (TIFF 3048 kb)

Supplementary material 7 (DOCX 32 kb)

## Abbreviations

ID: Intrinsically disordered
MoRF: Molecular recognition feature
MoRE: Molecular recognition element
SLiM: Short linear motif
LCA: Last common ancestor
HBS: Helicase binding site
EBS: Enolase binding site
PBS: PNPase binding site
MTS: Membrane targeting sequence
CB: Composition bias
CD: Conserved domain
RISP: Region of increased structural propensity
