# Supplementary figures and images for: RNase E in the γ-Proteobacteria: conservation of intrinsically disordered noncatalytic region and molecular evolution of microdomains
Source: Mol Genet Genomics. 2014 Nov 29;290(3):847–62. doi: 10.1007/s00438-014-0959-5 (PMC4435900; doi:10.1007/s00438-014-0959-5)

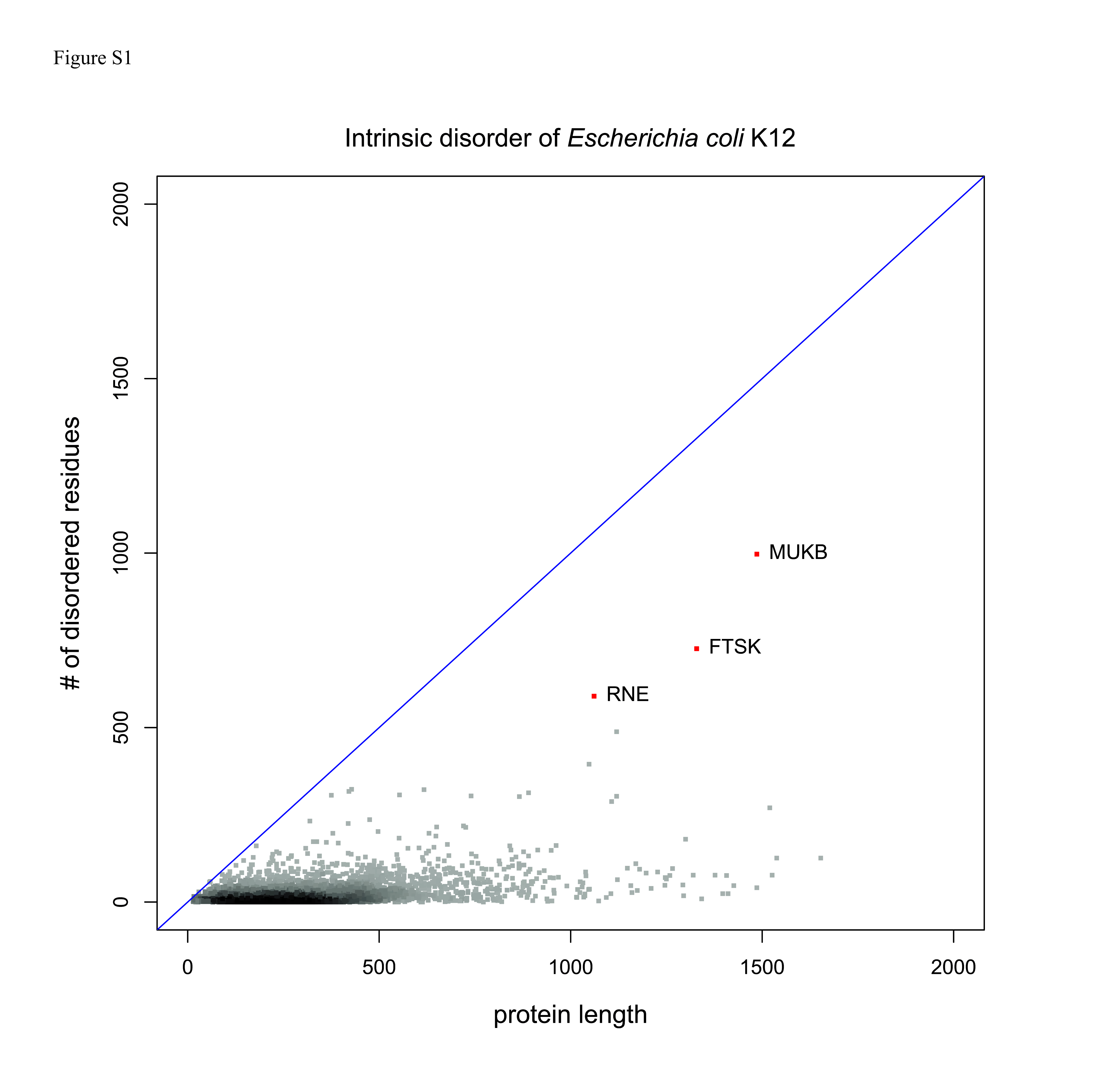

Supplement: Supplementary file 1 — Supplementary material 1 (TIFF 1047 kb) [file 438_2014_959_MOESM1_ESM.tif]

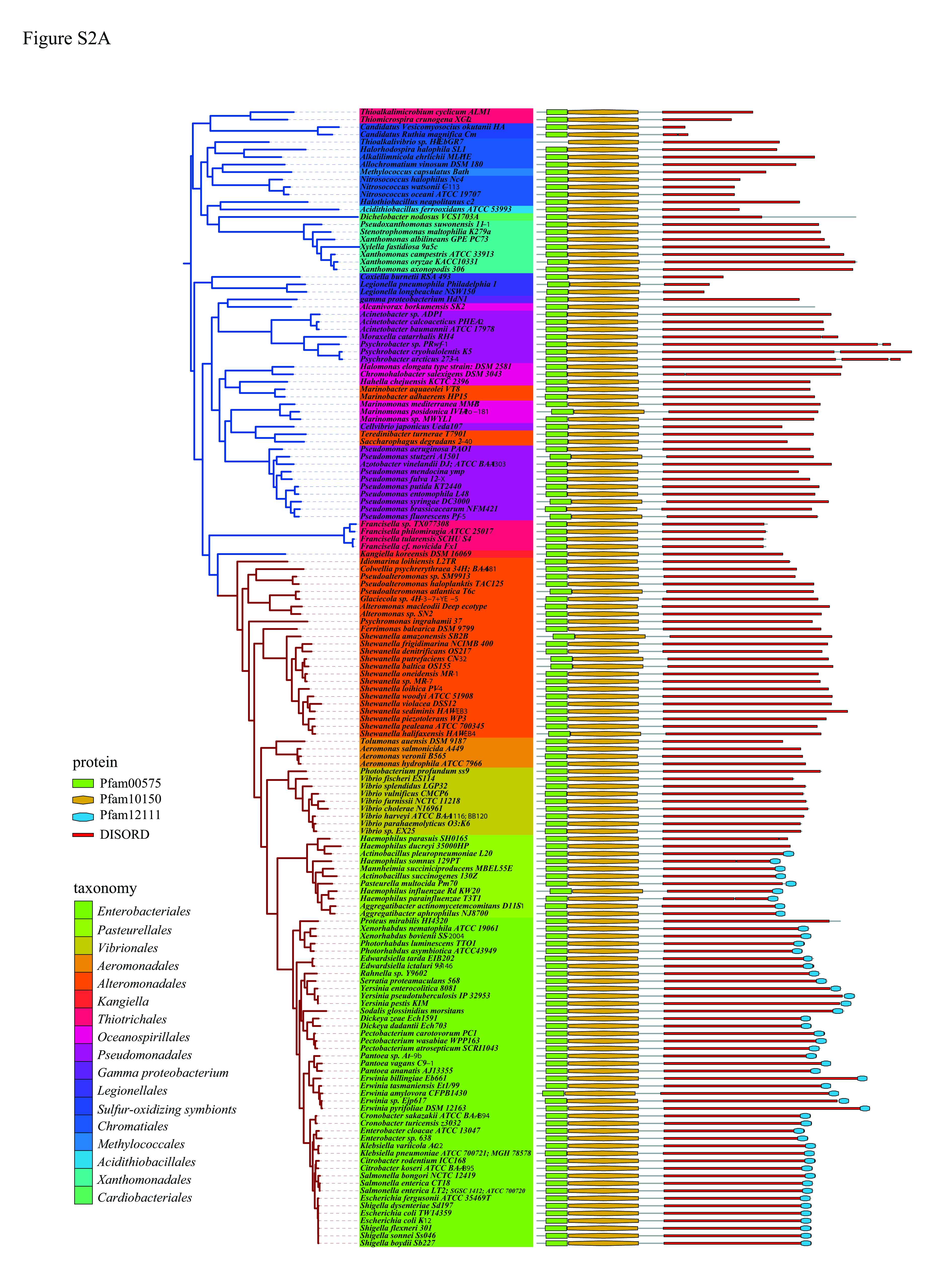

Supplement: Supplementary file 2 — Supplementary material 2 (TIFF 2837 kb) [file 438_2014_959_MOESM2_ESM.tif]

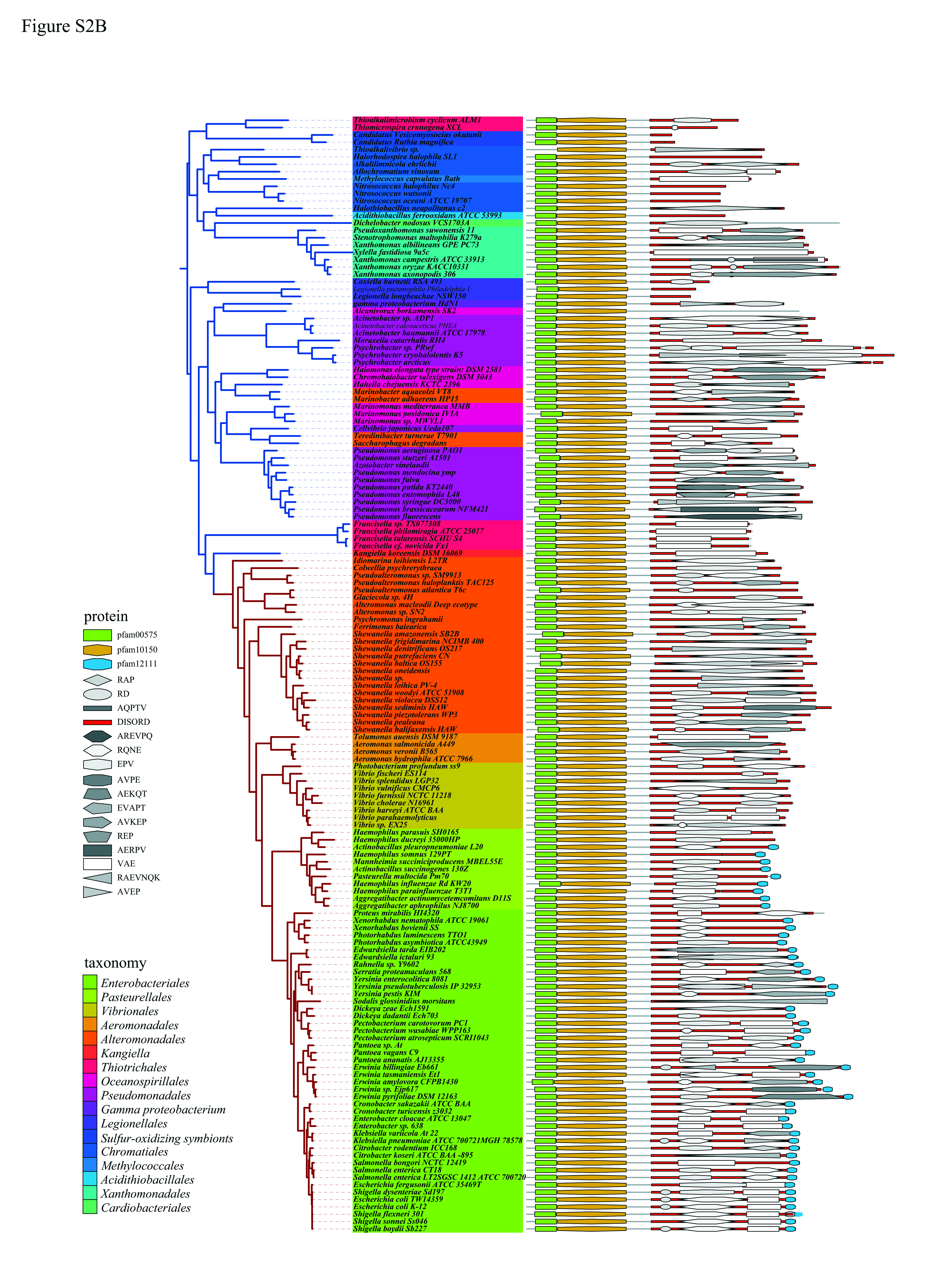

Supplement: Supplementary file 3 — Supplementary material 3 (TIFF 3259 kb) [file 438_2014_959_MOESM3_ESM.tif]

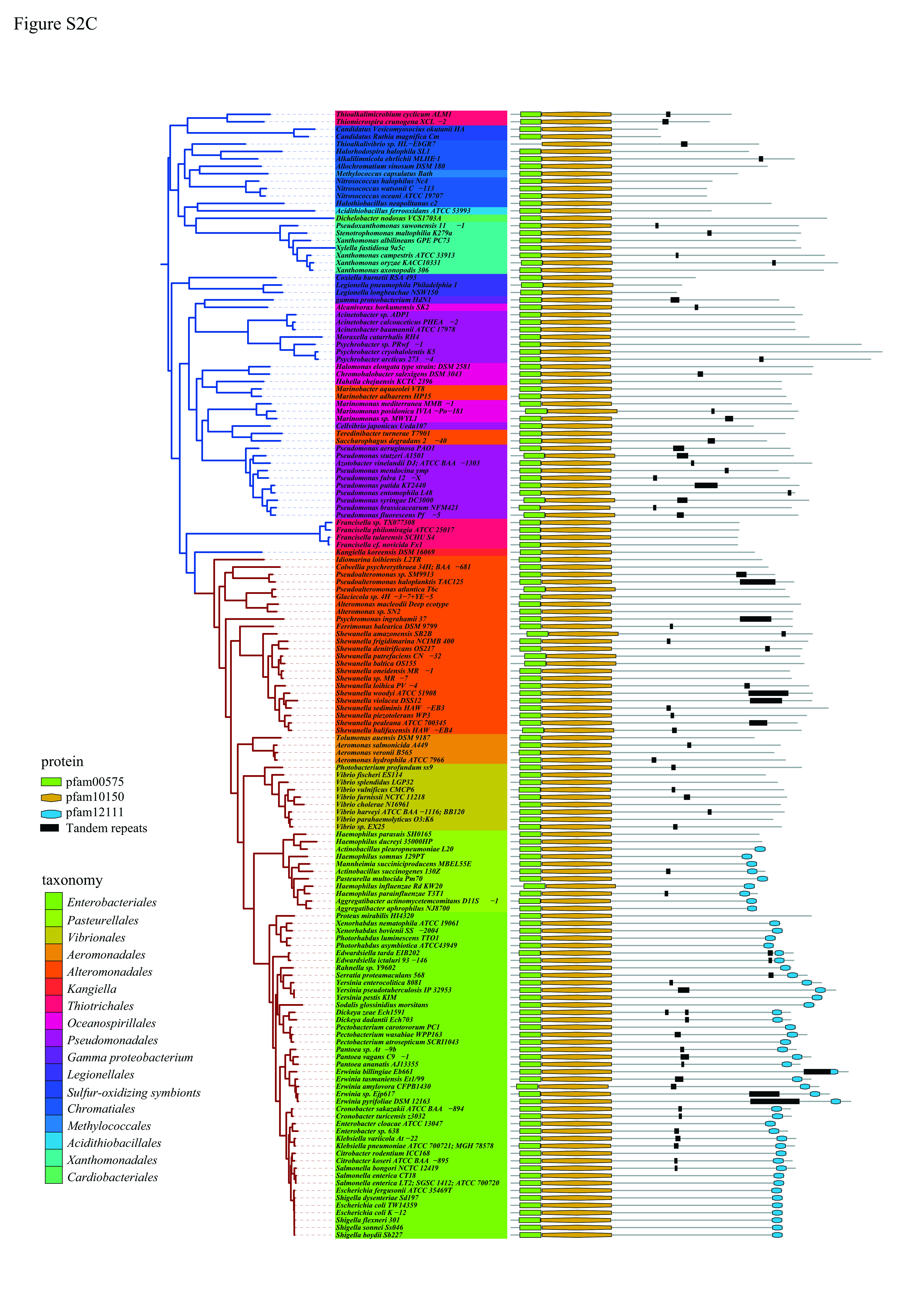

Supplement: Supplementary file 4 — Supplementary material 4 (TIFF 2772 kb) [file 438_2014_959_MOESM4_ESM.tif]

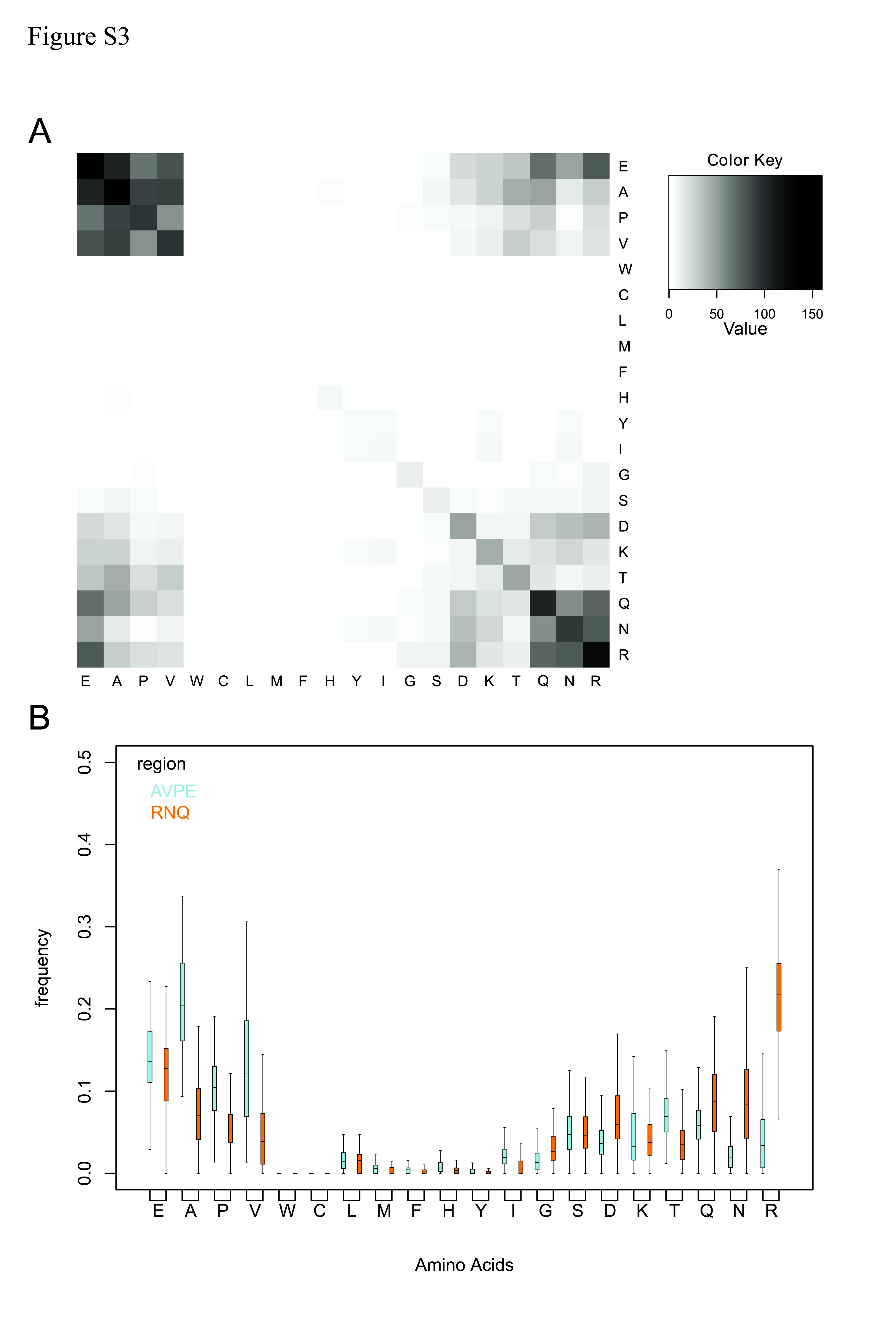

Supplement: Supplementary file 5 — Supplementary material 5 (TIFF 1154 kb) [file 438_2014_959_MOESM5_ESM.tif]

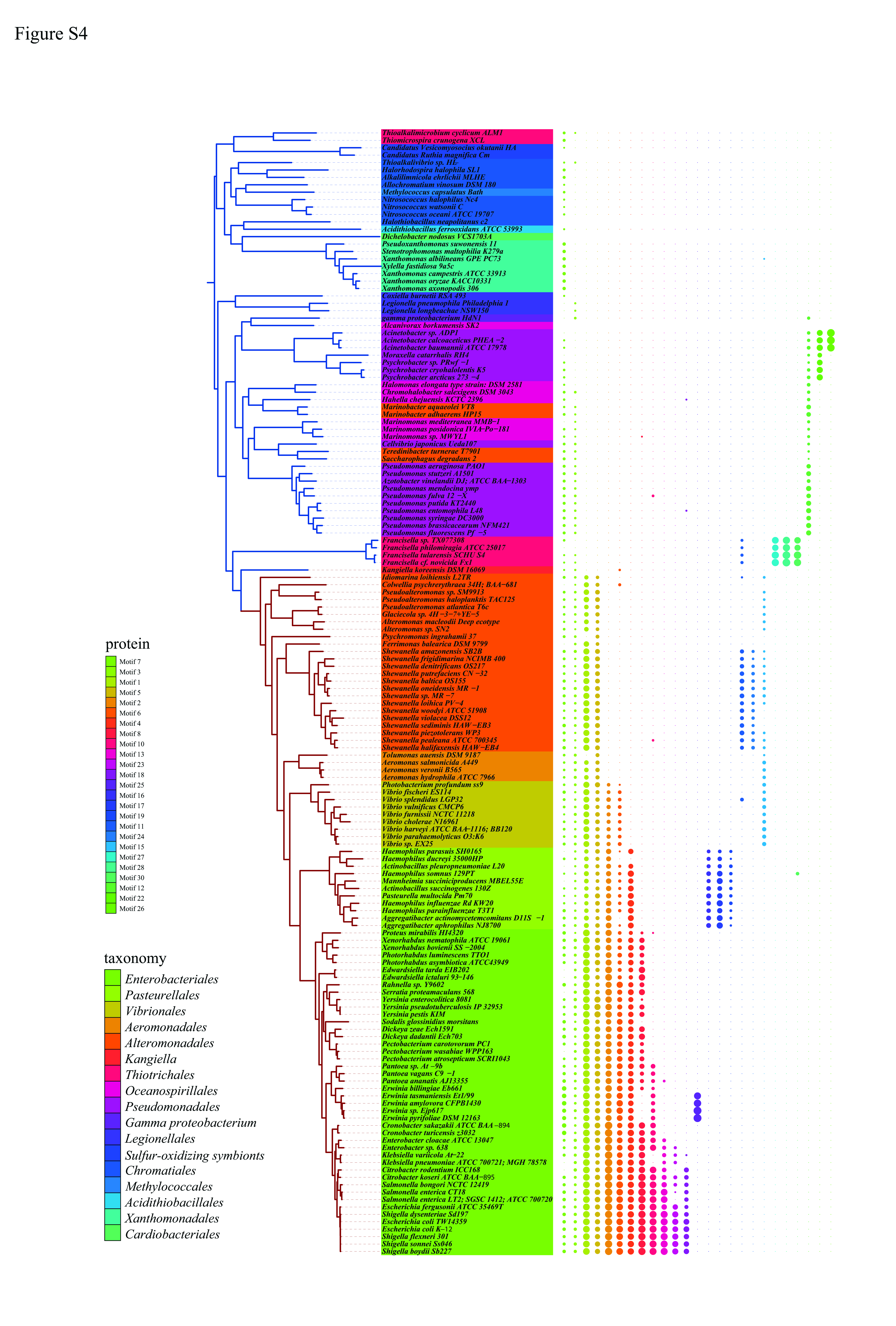

Supplement: Supplementary file 6 — Supplementary material 6 (TIFF 3048 kb) [file 438_2014_959_MOESM6_ESM.tif]
