# Supplementary material for: RNase E in the γ-Proteobacteria: conservation of intrinsically disordered noncatalytic region and molecular evolution of microdomains
Source: Mol Genet Genomics. 2014 Nov 29;290(3):847–62. doi: 10.1007/s00438-014-0959-5 (PMC4435900; doi:10.1007/s00438-014-0959-5)
Supplement: Supplementary file 7 — Supplementary material 7 (DOCX 32 kb) [file 438_2014_959_MOESM7_ESM.docx]

**SUPPLEMENTARY INFORMATION**

**Submission to *Molecular Genetics and Genomics* Article**

**RNase E in the γ-Proteobacteria: conservation of intrinsically disordered noncatalytic region and molecular evolution of microdomains.**

Soraya Aït-Bara^1, 2^, Agamemnon J. Carpousis^1^* and Yves Quentin^1^

^1^Laboratoire de Microbiologie et Génétique Moléculaires, UMR 5100, Centre National de la Recherche Scientifique & Université Paul Sabatier, 31062 Toulouse, France

^2^Current address: Microbes, Intestin, Inflammation et Susceptibilité de l'Hôte, Institut National de la Santé et de la Recherche Médicale & Université d'Auvergne, 63001 Clermont-Ferrand, France

*Corresponding author: LMGM, UMR5100, CNRS & Université Paul Sabatier, 118, route de Narbonne, 31062 Toulouse Cedex 9, France

Agamemnon.Carpousis@ibcg.biotoul.fr
+33561335972

**Table S1**. Tandem repeat prediction in the noncatalytic region of RNase E orthologs using XSTREAM. Only repeats with a period of at least 7 amino acids are shown. The consensus sequences are displayed below the alignment.

| **Strain** | **RNase E** | **Positions** | **Period** | **Copy Number** | **Consensus Error** | **Sequence alignment** |
| --- | --- | --- | --- | --- | --- | --- |
| *Shewanella violacea* DSS12 | RNE | 906-1025 | 28 | 4.29 | 0.14 | **EAQSASAAPTKPAAPVQ-AETQVKVEAK-**  **-AQSASAAPAKPAAPVQ-------VEAKA**  **EAQSASAAPTKPAAPVQTE-APMKVEVKA**  **EAQSASAAPTKPAAPVQTA-APVKVEAKA** **EAQSASAAPAKTADPV**  =============================  **EAQSASAAPTKPAAPVQTA-APVKVEAKA** : : : : ::::::: : : |
| *Shewanella sediminis* HAW-EB3 | ABV37241.1 | 951-1063 | 24 | 4.46 | 0.19 | **APVKAEASV-A-SAAPAK---PA---TPVKAE**  **TQVKTEASV-A-AAAPAK---PA---VQVKAE**  **APVKTEASV-A-TAAPAKPVAPAKVEASVKAE**  **APVKAKASV-A-AAAPAK---PA---APVKAE** **APVETE--VPAKA**  ================================  **APVKTEASV-A-AAAPAK---PA---APVKAE** :: ::::: : :: ::: ::::: |
| *Pseudomonas mendocina* ymp | ABP84383.1 | 608-671 | 21 | 3.05 | 0.19 | **R--RDDERK-PREERAPREERQA-**  **REPR-EERQ-PREERAPREER-AP**  **REPR--EGQENRRERKPREER-AP** **RE**  ========================  **REPR-DERQ-PREERAPREER-AP** :: :* :::: : : : : |
| *Ferrimonas balearica* DSM 9799 | ADN75591.1 | 960-1023 | 17 | 3.76 | 0.09 | **EEVKTEVTEAPAEEVV-A**  **EEVKAEVSEAPAEE-AVA**  **EEVKAEVTEAPAEEVA-A** **EEVKAEVTEAPVE**  ==================  **EEVKAEVTEAPAEEVA-A** : : : ::: |

| *Shewanella piezotolerans* WP3 | ACJ29773.1 | 951-1014 | 16 | 3.94 | 0.09 | **EAVKAEPVAEAEAPVKT**  **EAVKTEP-AEAKAPVKT**  **EAVKAEP-VEAKAPVKT**  **EAVKAKP-AKAKAPVK**  =================  **EAVKAEP-AEAKAPVKT** :: ::: : |
| --- | --- | --- | --- | --- | --- | --- |
| *Shewanella woodyi* ATCC 51908 | ACA86316.1 | 900-1049 | 15 | 10.00 | 0.11 | **KPEVSVKTEA-T-AAPA**  **KSEAPVKAEA-T-SAPT**  **KPETPVKAEA-T-SAPT**  **KPETPVKAAA-T-SAPT**  **KPEAPVKAEA-T-SAPT**  **KSEAPVKAAA-T-SAPT**  **KPEAPVKAAA-T-SAPT**  **KPEAPVKAAA-T-SAPT**  **KPEAPVKAAA-T-SAPT** **KPEAPVKAEASTASA**  =================  **KPEAPVKAAA-T-SAPT** : :: :: : :: : |
| *Shewanella halifaxensis* HAW-EB4 | ABZ76332.1 | 873-999 | 13 | 9.15 | 0.08 | **EAP---V-VQAPAEVKV**  **EAPVASVSVETPAEVKV**  **EAPVASVPVETPAEVKV**  **EAP---V-VETPAEVKV**  **EAP---V-VETPAEVKV**  **EAP---V-VETPAEVKV**  **EAP---V-VETPAEVKV**  **EAP---V-VETPAEVKV**  **EAP---V-VETPAEVKV** **EA**  =================  **EAP---V-VETPAEVKV** ::: : :: |

| *Erwinia billingiae* Eb661 | RNE | 1110-1210 | 13 | 7.77 | 0.07 | **PVEAPVALTPVAA**  **PVEAPVAQAPVAT**  **PVEAPVAQAPVAA**  **PVEAPAAQAPVAA**  **PVEAPVAQTPVAA**  **PVEAPVAQAPVAA**  **PVEAPAAQAPVAA** **PVEAPAAQAP**  =============  **PVEAPVAQAPVAA** : :: : |
| --- | --- | --- | --- | --- | --- | --- |
| *Pseudoalteromonas haloplanktis* TAC125 | RNE | 868-1000 | 12 | 11.08 | 0.08 | **TEEPAKVETPVV**  **TEEPAKVETPVA**  **AEEPAKVETPVA**  **AEEPAKVEAPVV**  **TEEPAKVETPLV**  **TEEPAKVEAPVV**  **TEEPAKVETPVV**  **TEEPAKVEAPVV**  **TEEPTKVETPVV**  **TEEPTKVEAPVV**  **TEEPAKVETPVV** **T**  ============  **TEEPAKVETPVV** : : : :: |
| *Pseudomonas entomophila* L48 | CAK14467.1 | 614-649 | 12 | 3.00 | 0.15 | **EERKPREE---R**  **NERAPREERQPR**  **EERAPREERAPR** **EER**  ============  **EERAPREERAPR** : : :*: |

| *Aeromonas hydrophila* ATCC 7966 | ABK38103.1 | 664-697 | 11 | | 3.00 | 0.19 | **EREPREA-REPR-Q**  **EREPR-A---PRPA**  **-REPR-ASREPR-A** **E**  ==============  **EREPR-A-REPR-A** : : ::: :: |
| --- | --- | --- | --- | --- | --- | --- | --- |
| *Erwinia tasmaniensis Et1/99* | AMS | 640-673 | 11 | | 3.09 | 0.11 | **DRNERGAERNT**  **DRNERSAERNT**  **DRNERSNERN-** **ER**  ===========  **DRNERSAERNT** : :: : |
| *Shewanella pealeana* ATCC 700345 | ABV87824.1 | 904-969 | 10 | | 6.40 | 0.15 | **PVAKPEVE--AK**  **PVVEPIVE--AK**  **PVVEPIVE--AK**  **PVVEPTVE--AK**  **PAVEPTVE--AK**  **PVVEPTVEVKAK** **PS-EP**  ============  **PVVEPTVE--AK** ::: : :: |
| *Erwinia tasmaniensis* Et1/99 | AMS | 622-652 | 10 | | 3.00 | 0.19 | **GER-TERNADR**  **GER-NDRNADR**  **NERGAERNTDR**  ===========  **GER-AERNADR** : :*: : |
| *Pseudomonas aeruginosa* PAO1 | RNE | 616-655 | 9 | | 4.44 | 0.15 | **PREERAERQ**  **PREERAERP**  **NREERSER-**  **RREERAERP** **AREER**  =========  **PREERAERP** * : : |
| *Ferrimonas balearica* DSM 9799 | ADN75591.1 | 669-700 | | 9 | 3.56 | 0.06 | **REERRDDSR**  **REERREESR**  **REERRDDSR** **REERR**  =========  **REERRDDSR** :: |
| *Saccharophagus degradans* 2-40 | ABD80881.1 | 746-772 | | 8 | 3.38 | 0.00 | **QAKSEAKE**  **QAKSEAKE**  **QAKSEAKE** **QAK**  ========  **QAKSEAKE** |
| *Thiomicrospira crunogena* XCL-2 | ABB41299.1 | 575-596 | | 7 | 3.14 | 0.13 | **N-NRNNN**  **NRNRNNN**  **NRNRNNR** **RR**  =======  **NRNRNNN** :: : |
| *Erwinia pyrifoliae* DSM 12163 | RNE | 620-646 | | 7 | 3.86 | 0.18 | **RNGDR-NE**  **RN-DRSGE**  **RN-DRSAE** **RN-ERSA**  ========  **RN-DRSAE** :: :: |
| *Erwinia sp.* Ejp617 | RNE | 617-643 | | 7 | 3.86 | 0.18 | **RNGDR-NE**  **RN-DRSGE**  **RN-DRSAE** **RN-ERSA**  ========  **RN-DRSAE** :: :: |

**Supplementary Figure Legends.**

**Fig. S1.** Prediction of intrinsically disorder regions in *E. coli* K12 proteome.

DISOPRED2 with the threshold of false positive prediction was set at 5% was used to predict regions of intrinsic disorder. The number of disordered residues is plotted as a function of protein length. The diagonal line represents the limit at which a protein are 100% disordered. Most proteins with large disordered regions (greater than 50%) are small to average in length (less than 600 residues). RNase E, FtsK and MukB are very large proteins (greater than 1000 residues) that are more than 50% disordered.

**Fig. S2.** Intrinsic disorder, composition bias and repeat sequences in the noncatalytic region of RNase E orthologs.

In each panel, the primary structure of a representative selection of RNase E homologs (right half of panel) has been mapped to the species tree of the γ-Proteobacteria (left half of panel) constructed as described (Materials and Methods). The blue branches correspond to a subdivision that includes the PO clade (*Pseudomonadales* and *Oceanospirillales*); the red branches to the VAAP clade (*Vibrionales, Aeromonadales, Alteromonadales, Pasteurellales*) and *Enterobacteriales*. Tree leaves are color coded according to taxonomy (key). Symbols for Pfam domains are indicated in the protein key. **A.** Disordered regions (DISORD), **B.** Composition bias (protein key), and **C.** Tandem repeats. The N-terminal catalytic domain, which is a composite of the S1 RNA binding motif (Pfam00575) and the catalytic core (Pfam10150), is highly conserved. We verified with scan-for-matches program that all orthologs contained the CPxCxGxG motif corresponding to the Zn-link or a modified version in *Alkalilimnicola ehrlichii*, *Candidatus Vesicomyosocius okutanii*, *Candidatus* *Ruthia magnifica*, *Halorhodospira halophila*, *Xylella fastidiosa* and *Coxiella burnetii*. The small domain of the catalytic subunit for which there is no Pfam motif is also well conserved. RNase E orthologs in the *Enterobacteriales* and the *Pasteurellales* contain an additional domain (Pfam12111), which corresponds to the PBS1 of the *E. coli* homolog.

**Fig. S3.** Clustering of compositionally biased sequences in the noncatalytic region of RNase E homologs.

A. The gray scale is proportional to the frequency that the amino acid pair occurs in the compositionally biased region. The clustering suggest a high frequency and a strong association between A, E, P and V and between R, N and Q. Partial overlap is observed between both groups. The amino acids W, C, L, M, F, H, Y, I, G, S are underrepresented in the compositionally biased regions.

B. Amino acid frequencies were computed for all RNQ-rich and AEPV-rich regions predicted in our sample of sequences and reported in Figure 4. The distribution is summarized as a boxplot with results grouped by amino acid.

**Fig. S4.** Phylogenetic distribution of conserved sequence motifs in RNase E orthologs.

Sequence motifs in a representative selection of RNase E homologs (right half of panel) have been mapped to the species tree (left half of panel). Phylogenetic tree of γ-Proteobacteria species was constructed as described (Materials and Methods). The blue branches correspond to a subdivision that includes the PO clade (*Pseudomonadales* and *Oceanospirillales*); the red branches to the VAAP clade (*Vibrionales, Aeromonadales, Alteromonadales, Pasteurellales*) and *Enterobacteriales*. Only motifs with a taxonomic signal are displayed. A circle is drawn if at least one occurrence of the motif is found. The size of the circle is inversely proportional to the p-value of the best corresponding motif in the RNase E sequences. Motifs with low p-value (small circle) present a dispersed distribution. All motifs were predicted to be acquired once with a few losses in tree tips or recent subtrees. Only motif 15 shows acquisition followed by a clear loss; it could have been replaced by motif 4. Motif 3 (MTS) is not conserved in the upper part of the tree if filtered with a p-value of less than 1.0 e-6, but related sequences are found with a threshold p-value of less than 1.0 e-4. This result is probably due to an under representation of genomes of these species (i.e. fewer complete genome sequences available). Tree leaves are color coded according to the taxonomy (key). Motifs are color coded as indicated in the protein key.
